# Strengthening routine health data analysis in Ethiopia: the Operational Research and Coaching for Analysts (ORCA) experience

**DOI:** 10.1080/16549716.2021.1901390

**Published:** 2021-03-31

**Authors:** Joanna Busza, Seblewengel Lemma, Annika Janson, Serawit Omar Adem, Della Berhanu, Atkure Defar, Lars-Åke Persson, Carina Källestål

**Affiliations:** a Centre for Evaluation, Department of Public Health, Environment & Society, London School of Hygiene & Tropical Medicine, London, UK; b Department of Disease Control, Faculty of Infectious and Tropical Diseases, London School of Hygiene & Tropical Medicine, London, UK; c Department of Women´s and Children’s Health, Karolinska Institutet, Stockholm, Sweden; d Independent Consultant, Addis Ababa, Ethiopia; e Health System and Reproductive Health Research Directorate, Ethiopian Public Health Institute, Addis Ababa, Ethiopia

**Keywords:** Stig Wall, Capacity development, routine health information systems, Ethiopia, mixed methods, process evaluation, data quality

## Abstract

Many routine health information systems (RHIS) show persistent gaps between recording and reporting data and their effective use in solving problems. Strengthening RHIS has become a global priority to track and address national health goals. In Ethiopia, the Ministry of Health and Bill & Melinda Gates Foundation introduced the Operational Research and Coaching for Analysts (ORCA) capacity development project, co-designed with the London School of Hygiene & Tropical Medicine, which delivered training, coaching and mentoring support. We present the development, experiences, and perceptions of ORCA as a mechanism to enhance data quality, analysis, interpretation and use. ORCA integrated capacity development activities into national data analysts’ routine workload over a period of 2 years. Participating analysts were drawn from across the Ministry of Health directorates and two of its closely aligned agencies: the Ethiopian Public Health Institute and the Ethiopian Pharmaceutical Supply Agency. We used mixed methods (knowledge questionnaire, semi-structured interviews, programme records) to document the fidelity, feasibility, reach, and acceptability of ORCA and identify early signs of improved knowledge and changing institutional practices. Thirty-six participants completed the programme. Working in interdisciplinary groups on specific national health indicators, they received training workshops and support for study design, fieldwork, and analysis to build skills in assessing data quality and interpreting findings relevant to policy. Personal development grants and laptops provided incentives for sustained engagement. Participants appreciated ORCA’s applied and practical approach as well as good communication from administrators and clear links to national strategy. They also expressed frustration with delays, difficulties prioritising project work over routine responsibilities, and lack of formal accreditation. Knowledge and analytic skills increased and participants were able to integrate experiences from the project into their future work. Health system managers saw potential in longer-term improvements in data analysis and application to policy, although no clear changes were observed yet.

## Background

The United Nations Development Programme defines capacity development as ‘*the process through which individuals, organizations and societies obtain, strengthen and maintain the capabilities to set and achieve their own development objectives over time’* [1]. Routine health information systems (RHIS) are one part of the health system that has become a global priority for capacity development initiatives as a means to improve and monitor national health outcomes and goals [2,3]. RHIS in many low-income countries remain weak and underutilised for evidence-based decision-making [4]. In response, governments and donors recommend efforts to improve RHIS quality focused on data collection, use, and processes [5].

Health system improvements are only sustained once embedded into institutional culture, processes and practices so that they are resilient to changes in leadership, staffing and policies over time [6]. Strengthening capacity is multi-dimensional, comprising a mix of technical assistance, training and skill-building, new operational tools, peer networking, and incentives. It is multi-level, targeting national health architecture, organisational management, and frontline staff performance. RHIS capacity strengthening frequently centres around new technologies (e.g. the widely adopted DHIS-2) and technical skills (data entry, transfer, aggregation, synthesis, analysis, and interpretation). Capacity strengthening often consists solely of training, lacking deeper engagement with whole health systems [7]. Few capacity development efforts are evaluated [8], although evidence is accumulating to suggest that they are most likely to succeed if they involve genuine country ownership and investment in the process, utilise problem-based ‘on the job’ learning, and embed technical assistance within existing health system structures and practices [6,9,10].

Ultimately, the need for RHIS capacity strengthening is highlighted by persistent gaps between recording and reporting information and its applied use in solving problems [4]. Functioning RHIS systems should support action rather than data analysis for its own sake, so that each type of data collected has a clear purpose and is appropriately interpreted to inform policies and programmes that improve health outcomes at population level [11].

Ethiopia has put RHIS quality at the forefront of its health agenda. Recognising serious problems of routine data quality [12–14], the Government of Ethiopia included strengthening RHIS, in Ethiopia called the routine Health Management Information System (HMIS) within its ‘Information Revolution’ in the first Health Sector Transformation Plan of Ethiopia [15]. The Ethiopian Ministry of Health (MOH), prioritised capacity strengthening of analysts in its agencies, identifying this public health cadre as central to effective use of national routine HMIS data. In response, the Operational Research and Coaching for Analysts (ORCA) training and mentoring project were conceived by the MOH’s Policy & Planning Directorate (PPD) and Bill & Melinda Gates Foundation (BMGF), which provided funding. ORCA was co-designed and finalised in partnership between the MOH, BMGF and the London School of Hygiene & Tropical Medicine (LSHTM), which delivered training, coaching and mentoring support.

In this paper, we present the development, implementation, experiences, and perceptions of the ORCA training and mentoring project. We reflect on the project’s design and practice as a case study with the potential to contribute to improved understanding of the facilitators and barriers to strengthening RHIS system functioning in low-income settings.

### ORCA development

The ORCA project was devised as a means to integrate capacity development within national data analysts’ routine workload. Data analysts are situated across all MOH directorates as well as in two of its closely aligned agencies, the Ethiopian Public Health Institute (EPHI) and the Ethiopian Pharmaceutical Supply Agency (EPSA). EPHI and EPSA are tasked with collecting, synthesising, analysing, and presenting data from national RHIS sources to policymakers. The MOH stipulated that participants of the ORCA project should remain full-time employees at their respective institutions, and that activities should avoid interfering with their daily workload. Rather, it was expected that ORCA would play a synergistic role, supporting analysts to develop skills they could apply to their routine work, and encouraging them to overcome vertical working structures and collaborate more closely with counterparts across health agencies.

LSHTM established a research office within EPHI in 2015 from which it conducts studies in collaboration with EPHI and MOH staff primarily on maternal and child health service coverage and quality. It was thus well placed to implement the ORCA project continuously over 2 years. The stated aim of ORCA was ‘to support and strengthen the capacity of analysts at the Ministry of Health to perform and report high-quality analyses of key health metrics that can inform decision-making’. ORCA brought together staff from the three main Ethiopian health system agencies and across directorates into interdisciplinary working groups arranged around key health themes. This intended to build alliances and collaboration across agencies that often operated vertically with little coordination.

ORCA incentivised participation through both individual (professional development grant) and group-based (small operations research budget) financial incentives as a means of increasing participants’ motivation and retention during their participation in ORCA, considering the additional work burden incurred [16]. As part of institutional commitment to the project, each participating health agency continued to pay the participating staff’s salary and agreed to their attendance in ORCA activities during work hours.

**Figure 1.**
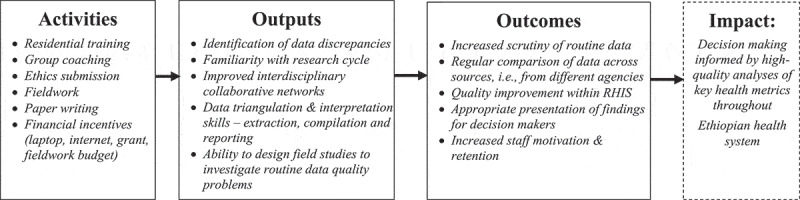
ORCA capacity development model

ORCA’s design drew on theoretical approaches from adult education, which prioritise group-based problem-solving and ‘experiential learning’, where new skills are introduced in the context of routine work practice [17,18]. These were hypothesised to support a process of normalisation [19], producing work cultures that valued and encouraged routine scrutiny of data quality and efforts to improve it, with the outcomes of higher quality data and more effective analysis and use (Figure 1).

We combined residential workshops with small-group coaching, following an operational research cycle to explore extent and reasons for poor data quality within Ethiopia’s RHIS.

Box 1. summarises all capacity building components included in ORCA.

**Table:** 

Specific aims To foster critical thinking regarding data quality, including validation and triangulation of data across sources To increase interdisciplinary discussions on how to reduce the presence of bias in key health metrics that are reported and used for planning and policy formation To incubate and promote the retention of an internal team of experts who will drive improvements in the generation, management, and use of data within the Ethiopian health system. To generate improved and more robust measurements on an agreed set of routine health information management indicators. Recruitment of participants Participation was competitive. The opportunity was circulated throughout directorates at MOH, EPHI and EPSA that directly handle routine health information and employ analysts. The application included a short data analysis and interpretation test accompanied by questions on candidates’ experience and motivation for participating. The number of participants was capped at 40, and quotes were set at 20 from MOH and 10 each from EPHI and EPSA, with 50% male/female distribution. Oversight and Staffing ORCA management team included a principal investigator, contracted by LSHTM and administrator and IT-lead contracted by WHO. Academic staff included ‘coaches’, who were experienced researchers and PhD:s. A Technical Advisory Group (TAG) ensured alignment with MOH strategy and consisted of directors from the 3 government agencies and ORCA leadership and was chaired by an MOH directorate head. The TAG was tasked with monitoring ORCA progress, suggesting modifications and liaising between ORCA and relevant MOH bodies. Financial and Material incentives The programme budgeted included a grant (USD 10,000) and personal laptop for each individual participant as an incentive to apply to the ORCA project and contribute free time to its deliverables. To reduce attrition of staff during the programme, the grant was paid in 4 instalments based on deliverables. There was a further budget to finance fieldwork costs during the mini-studies conducted by each small group.	Thematic Group Work Selection of six thematic areas that formed the basis of small working groups to which all participants were allocated for the remainder of the programme: *Maternal Health, Neonatal Survival, Child Nutrition, Immunization, Malaria and Tuberculosis*. Groups were organised to ensure diversity by discipline, agency and directorate, as well as to have a genderbalanced composition. Groups worked together throughout organised workshops and between them during their own time. Residential training workshop A combination of 3- and 5-day training workshops were organised outside of Addis Ababa to facilitate group interaction and reduce office-related distractions. Workshops provided intensive training and practicals covering: R-software, study protocol design, qualitative methods and analysis, writing up mixed method studies for dissemination. Research Proposal Each thematic group developed a research proposal for submission to the EPHI Institutional Review Board for ethical approval. Two objectives were adapted for each group as follows: a) to analyse regional and national existing data using the concepts of outliers, international consistency, consistency between related indicators and external consistency between data sources. b) to analyse facility level data and use, applying quantitative and qualitative methods to explore reasons for data quality issues at regional and district levels. Data Collection and Analysis All groups planned and conducted field studies conducted over 6–11 days with a shorter pilot phase during a feasibility visit. Each group gathered district level quantitative and qualitative data. For the first study objective teams conducted shared analysis. For the second objective groups analysed data based on tailored plans identified during protocol development. Coaching and Mentorship Each group was assigned an academic team member for fortnightly supervisory meetings to discuss progress during the applied work, accompanied by regular email support. Other LSHTM mentors participated in training workshops to deliver key skill-building components (e.g. qualitative analysis, writing support).

## Methods

As part of our case study, we adopted mixed methods drawn from process evaluations to prospectively document the intervention. We ensured we could capture *fidelity* to design (were activities conducted as planned), *feasibility* of delivery (what challenges were faced and how were they addressed), *reach* (how many people participated throughout from intended target groups), and *acceptability* to participants and key stakeholders (how were activities received by those directly involved). We also aimed to capture early signs of intended outputs and outcomes. We did not attempt to measure impact level change, given the short time frame. We used both quantitative and qualitative methods (Table 1).

**Table 1.** ORCA monitoring framework

Measure	Documentation
Fidelity	Schedule of activities Project narrative kept by ORCA staff, including reflections on implementation progress Annual reports
Feasibility	Project narrative kept by ORCA staff, including reflections on implementation progress Semi-structured interviews with key health system stakeholders
Reach	Application records Attendance registers
Acceptability	Semi-structured interviews with participants at two time periods Semi-structured interviews with key health system stakeholders at two time periods
Plausible effect	Pre- Mid- and Post- survey using the Evidence Based Practice questionnaire Semi-structured interviews with participants and directorate managers at two time periods Number and quality of outputs demonstrating improved data interpretation and presentation Retention of participants within their work

We kept narrative accounts of all ORCA activities, including delays and deviations from the original timing and structure. Routine monitoring data were collated and included project records of recruitment, retention and attendance.

An independent researcher (SOA) conducted semi-structured qualitative interviews with eight participants and three managers (one per health agency) 12–15 months into the project. This researcher was not involved in the design or implementation of ORCA other than for qualitative data collection. She was provided with a list of all relevant stakeholders and the 38 participants and purposively selected participants to ensure diversity across sex, role and agencies. All three managers and 7 out of 8 sampled participants were interviewed a second time within 2 months of project completion. The topic guide explored participants’ reasons and expectations when joining the project, benefits and challenges experienced during ORCA, and opinions of its usefulness and quality, including suggestions for improvement. Among stakeholders it addressed expectations and experiences, views of the project’s overall value, and recommendations. The first round of interviews was conducted in person and lasted 30–60 minutes. Follow-up interviews built on findings from the first interview round, and probed about changes in perceptions; these were conducted by phone due to the SARS-CoV-2 pandemic. The independent researcher recorded and transcribed interviews directly into English, removing identifying information. Two ORCA staff conducted thematic content analysis by first reading each transcript multiple times for familiarisation, and applying an agreed coding framework comprising both deductive and inductive codes to each transcript. Comparisons were made across participants, their agencies and position in the organisation. Illustrative quotes were identified solely by role (participant or manager) and gender of participants to reduce the likelihood of inadvertent identification.

We administered the validated Evidence-Based Practice (EBP) Questionnaire adapted for the health sector [20] at the first workshop (June 2018), at mid-term (September 2019) and at project end, due to the SARS-CoV-2 pandemic by a web-based questionnaire (June 2020). Using self-reported data, the EBP measures change in use of evidence in routine professional practice, specifically attitudes toward relevance and value of EBP, knowledge of terminology, and confidence and frequency of integrating EBP into their work. Frequencies and proportions were calculated and differences between males and females and between MSc qualified and not MSc qualified, respectively, were assessed using Mann-Whitney-Wilcoxon tests.

## Results

Findings are presented chronologically, following the change pathway from implementation of activities through outputs to indication of effect at outcome level.

### Enrolment and participation

In total, 138 analysts applied, of whom 98 were eligible and submitted full applications that included data analysis and interpretation exercises based on real routine data. Applications were scored by four ORCA staff following specified criteria. Forty participants were selected based on scores and quotas to ensure proportional distribution by agency and sex. The MOH introduced an additional requirement for its employees to sign a ‘training agreement’ obliging them to remain in their current posts for 8 months. This requirement dissuaded two successful candidates from enrolling.

All 38 participants (23 males and 15 females) attended the launch meeting. A list of themes was suggested by the MOH from which participants selected topics of interest. Maternal Health, Neonatal Survival, and Child Nutrition groups began in July 2018. Immunization, Malaria and Tuberculosis started 1.5 months later. All groups attended seven residential training workshops, each lasting three to 4 days: ‘Planning and Problem Formulation’; ‘Research Methodology’; ‘Using R Commander’; ‘Mixed Methods Analysis;’ ‘Analysing Regional and National Data’; ‘Preparing Field Study Analysis’ and ‘Preparing for Dissemination of Results’. The final workshop was held online due to the SARS-CoV-2 pandemic.

The participants attended a median of 67% of planned group meetings (range 25–100%). Meetings were frequently rescheduled because members did not complete their allocated tasks or could not spare the time due to last minute work conflicts. At each training workshop, there were 2–14 participants absent.

Individual grants of, in total, USD 10,000 USD per participant were disbursed in four instalments based on deliverables, including meeting protocol deadlines, completing field studies, and workshop attendance. Only one participant did not receive the second instalment due to absenteeism. The third and fourth instalments were not received by two participants who relocated to other countries and thus left the project, making 36 participants complete the programme.

### Implementation challenges

ORCA experienced several delays over its 2 years, particularly during the start-up phase as it took 6 months to formalise the partnership. Each thematic group was meant to obtain ethical clearance for their fieldwork proposal in the first year, with fieldwork in early 2019. For many participants, reading the literature, designing the project, developing data collection tools and writing in scientific English following a pre-designed format proved a larger undertaking than envisioned. Meetings with coaches lasted two to 3 hours and required six to 15 separate sessions to complete the proposal, with a median of 7.5 meetings per group. The ethical review took 6 weeks to 3 months. Once groups were ready to conduct fieldwork, a civil unrest in 2019 meant most groups postponed data collection to late in the year 2019.

In early 2020, during coaching for integrated data interpretation and presentation of results, the SARS-CoV-2 pandemic resulted in closure of all Ethiopian government offices. All coaching thereafter was conducted using online video conferencing. Nonetheless, the project culminated in the production of group reports, two joint publications (under review), and a policy brief. Participants presented posters on their findings at conferences.

### Participants’ views of implementation

The applied and practical approach of ORCA was appreciated, particularly the way the project was integrated within participants’ routine work responsibilities and designed to support their performance. Respondents appreciated the participatory and applied approach and the way coaching was tailored to the thematic groups’ needs:

Some of the coaching is interactive. We will practice it after they show us with doing first. I personally say that it is better than trainings and workshops that were given previously because it requires practical work. (Participant 8, female, round 1)

After [coaching] feedback, when you see what you have done before and see your gaps, you understand you must improve … they have [offered] something very impressive. … Their comments enable you to improve yourself, contribute something, develop a feeling of ownership [over your work] and see yourself as mature person - it helps you to feel responsibility. (Participant 7, male, round 2)

Respondents also praised the regular communication from ORCA administrators. Several respondents described how they received weekly updates including reading materials relevant to the ORCA programme. Information about logistics, timing of workshops and any changes were also seen to be conveyed efficiently, in good time, and in a friendly and supportive manner:

Workshops are prepared on time, during the workshop the facility and the logistics, cars are arranged, all these things are fine. Current updates are sent on time, the admin is good. We have to say it is perfect. (Participant 6, male, round 1)

Nonetheless, respondents expressed frustration with early delays, particularly related to the financial and material incentives.

In terms of structure, some felt the workshops were spread too thinly across the two-year project, causing a loss of momentum in the skills-building process as participants went back to their regular assignments between workshops.

Intervals between meeting times are very long. It is after you forgot a lot of things when we meet. … [It] causes you to forget a lot of things because you are learning while you are performing your regular job, you might forget so many things as you are engaged in your work. (Participant 6, male, round 2)

Furthermore, despite the agreed ‘on the job’ integrated model, not all participants were able to excuse themselves from other responsibilities and fully participate in activities. This caused tensions with other participants who felt saddled with working group tasks assigned to the absentee members:

All of us are busy with office duties. It is difficult to manage our time with our team [ORCA thematic group]. … For example, if I have scheduled to work with my team and if my boss says no, I have no option other than to miss the team work. This might even create personal conflict among the team. This should be considered seriously with Ministry of Health, EPHI and EPSA as they agreed to participate in the programme but sometimes, they say ‘no’ … It creates conflict … . (Participant 1, female, round 2)

The issue of formal accreditation was raised in several interviews. Given the length and intensity of ORCA, participants felt their efforts should be acknowledged through a recognised qualification or lead into more advanced opportunities:

It would be good if we got certified like as a Master’s program. … We have spent 2 years for this programme. … In other country you can achieve a Masters in one year. (Participant 2, male, round 2)

### Outputs: new knowledge, skills and work practices

At the start of ORCA, 37 of 38 participants completed the EBP questionnaire, dropping to 29 and 28 at midline and end, respectively. The differences between the three assessments were few; see Table 2. The participants considered evidence-based practice to be *relevant to their work* (61–63 out of a max of 70 in all assessments). *Sympathy for EBP* was also relatively high, with a mean score of 22–23 out of a maximum of 35 in all three assessments. *Knowledge of relevant terminology* increased over time with mean scores of 49, 54, and 64 out of a maximum of 85 at the three assessments, respectively. The mean score for self-reported *application of evidence-based practice within work*, however, remained consistently low, although it increased from 20 to 25 out of a maximum of 45. This was reinforced by the low-to-mid levels of *reported confidence* in performing evidence-based practice, which increased slightly from 36 of 55 at baseline to 38 at the midpoint and 44 at the end-line assessment. There were no significant differences observed between males or females or between educational levels.

**Table 2.** Results from the Evidence-Based Practice (EBP), Questionnaire distributed at start (June 2018), mid (September 2019) and end (June 2020) showing expressed relevance for EBP, sympathy for EBP, knowledge of EBP terminology, using EBP in daily work, and showing confidence in EBP activities among ORCA participants, and differences between males and females and MSc qualified and not MSc qualified, respectively

DOMAINS/SECTIONS (No of items, Scoring min-max)	SCORING 2018 mean (SD)	p-value*		SCORING 2019 mean (SD)	p-value*		SCORING 2020 mean (SD)	p-value*
Relevance (14 items,14–70)								
Total (n = 35, NA = 2)	60.7 (5.9)		Total (n = 29, NA = 0)	62.5 (4.7)		Total (n = 25, NA = 3)	63.2 (4.0)	
Males (21/35, NA = 1)	61.2 (6.0)	0.3322	Males (15/29)	61.9 (4.7)	0.3685	Males (17/25, NA = 0)	62.4 (3.8)	0.2929
Females (14/35, NA = 1)	59.3 (5.9)		Females (14/29)	61.9 (4.7)		Females (8/25, NA = 3)	64.9 (4.3)	
Masters (25/35, NA = 1)	59.9 (6.0)	0.3827	Masters (25/29)	62.2 (4.9)	0.2525	Masters (24/25, NA = 1)	63.0 (4.0)	0.3302
Other education (10/35, NA = 1)	62.2 (6.3)		Other education (4/29)	64.8 (1.0)		Other education (1/25, NA = 2)	67.0 (NA)	
Sympathy (7 items, 7–35)								
Total (n = 35, NA = 2)	21.9 (3.9)		Total (n = 26, NA = 3)	22.8 (4.7)		Total (n = 27, NA = 1)	22.9 (3.8)	
Males (21/35, NA = 1)	22.8 (3.9)	0.7955	Males (14/26, NA = 1)	23.1 (4.9)	0.6233	Males (17/27, NA = 0)	22.4 (4.2)	0.6138
Females (14/35, NA = 1)	21.4 (4.0)		Females (12/26, NA = 2)	22.6 (4.8)		Females (10/27, NA = 1)	23.7 (3.0)	
Masters (25/35, NA = 1)	21.6 (4.0)	0.2083	Masters (22/26, NA = 3)	23.3 (4.8)	0.1336	Masters (24/27, NA = 1)	23.0 (3.9)	0.35
Other education (10/35, NA = 1)	22.7 (3.9)		Other education (4/26, NA = 0)	20.5 (3.7)		Other education (3/27, NA = 0)	21.7 (2.5)	
Terminology (17 items, 17–85)								
Total (n = 33, NA = 4)	48.7 (9.6)		Total (n = 26, NA = 3)	54.3 (8.8)		Total (n = 26, NA = 2)	61.5 (10.6)	
Males (17/31, NA = 4)	45.8 (8.7)	0.103	Males (14/26, NA = 1)	50.2 (7.0)	0.03931	Males (16/26, NA = 1)	59.0 (9.2)	0.1323
Females (14/31, NA = 0)	51.6 (10.4)		Females (12/26, NA = 2)	59.0 (8.8)		Females (10/26, NA = 1)	65.4 (12.0)	
Masters (22/31, NA = 3)	49.0 (10.8)	0.8867	Masters (22/26, NA = 3)	54.6 (9.3)	0.7487	Masters (23/26, NA = 2)	60.2 (10.3)	0.0913
Other education (9/31, NA = 1)	46.3 (6.6)		Other education (4/26, NA = 0)	52.3 (7.8)		Other education (3/26, NA = 0)	71.3 (8.6)	
Practice (9 items, 9–45)								
Total (n = 32, NA = 5)	19.8 (6.5)		Total (n = 26, NA = 3)	24.6 (6.2)		Total (n = 25, NA = 3)	24.5 (5.7)	
Males (18/30, NA = 3)	17.9 (4.6)	0.06166	Males (12/26, NA = 3)	24.0 (6.0)	0.5884	Males (17/25, NA = 0)	23.7 (6.0)	0.9302
Females (12/30, NA = 2)	23.2 (8.1)		Females (14/26, NA = 0)	25.3 (6.5)		Females (8/25, NA = 3)	24.8 (5.4)	
Masters (22/30, NA = 3)	19.7 (7.0)	0.8272	Masters (22/26, NA = 3)	25.2 (6.4)	0.226	Masters (22/25, NA = 3)	24.2 (6.1)	0.4758
Other education (8/30, NA = 2)	19.9 (6.5)		Other education (4/26, NA = 0)	21.2 (2.3)		Other education (3/25, NA = 0)	22.3 (1.6)	
Confidence (11 items, 11–55)								
Total (n = 35, NA = 2)	36.0 (6.8)		Total (n = 28, NA = 1)	37.9 (5.9)		Total (n = 27, NA = 1)	43.5 (5.7)	
Males (20/34, NA = 0)	36.0 (7.4)	1	Males (15/28, NA = 0)	39.3 (4.7)	0.3799	Males (17/27, NA = 0)	42.2 (5.9)	0.1245
Females (13/34, NA = 1)	35.5 (6.2)		Females (13/28, NA = 1)	37.6 (5.7)		Females (10/27, NA = 1)	45.6 (4.9)	
Masters (24/34, NA = 1)	35.7 (7.2)	0.7893	Masters (24/28, NA = 1)	37.7 (5.7)	0.5101	Masters (24/27, NA = 1)	43.2 (6.0)	0.2156
Other education (10/34, NA = 0)	37.1 (6.6)		Other education (4/28, NA = 0)	39.3 (7.8)		Other education (3/27, NA = 0)	46.0 (1.0)	

* Mann-Whitney-Wilcoxon test.

During interviews, participants highlighted how they were able to integrate knowledge and skills from ORCA into their work, e.g. conducting new analyses or checking different sources of data for discrepancies more thoroughly than before:

Yes, I am implementing that in my normal work. Now when the data comes, I visualize, clean, do the first analysis by using R. … This is what I gained from ORCA. … [Although] the specific examples that they have presented [during ORCA] may not be directly related with your work, in fact the principles are similar whatever you do, but you have to customize it on your own work. (Participant 1, male, round 1)

It could be difficult to apply new skills and knowledge given the high workloads. When participants returned to their offices, they often had little control over daily routines and were not always in a position to think about how they might use what they had learned. Concerns were raised about sustainability of new practices, particularly as participants’ colleagues had not been exposed to the same training. Some participants felt they successfully could share new skills in the workplace while others were less confident their contributions would be valued:

I think I benefited as an individual, there is skill and knowledge, and the case team with which I am working benefited, and the directorate gained an input, this is what I think. (Participant 8, male, round 2)

We were five people from the [directorate] who participated in ORCA [but] they [managers/colleagues] never gather and ask each member of the team what everyone obtained and the benefit it has for our routine work (Participant 5, male, round 2)

### Outcomes: changing institutional behaviour

The ORCA initiative aimed to positively affect behavioural norms within the Ethiopian health system in the longer term. While participants may have gained new perspectives and capabilities for improving quality and use of routine data, we looked to senior health system managers for indication of whether they notice shifts in organisational culture in their respective directorates.

From the outset, managers demonstrated buy-in to ORCA’s model, emphasising the importance of both the embedded ‘on the job’ approach and provision of professional development incentives for individuals. The latter were flagged as particularly important given high staff turnover and difficulties retaining qualified staff throughout the health system:

What differentiates ORCA from other programmes is these people … get a small grant. … This small grant creates motivation and helps them continue their work in a good way. … Laptops are given for every participant. So rather than considering it as a routine task and getting bored, we have seen that they [participants] are better motivated to work hard. … This small grant helps build their capacity [and] bring new information into the health sector. (Manager 1, round 1)

We were able to retain our workers for about a year or longer, and along the way, they built their capacities. … Everyone is benefited, the organization is benefited (Manager 3, round 2)

The programme content’s usefulness for RHIS data analysis was also highlighted. Managers saw potential in longer-term improvement on data analysis and application to policy, although no clear changes were observed yet:

We are going to encourage them [ORCA participants] to mentor and train the people who are working under them. … It is a win-win situation because everyone benefits when they [staff] build their capacities and improve. (Manager 2, round 2)

It just made the participants [ORCA participants] become better performers in their routine jobs. … However, there was nothing I can specifically mention as impact brought to our organization after ORCA (Manager 3, round 2)

A manager suggested ORCA participants to be monitored over the next 6 months or more to gauge actual long-term retention and organisational effects. Reliance on just a small number of participants was also seen as inadequate to engender lasting change; managers suggested additional rounds of ORCA as well as decentralising it to regional and zonal levels.

## Discussion

The ORCA capacity building initiative was implemented in Ethiopia through a tripartite partnership between key MOH agencies, the BMGF and LSHTM. Over 2 years, 36 out of 40 intended participants completed the programme, which integrated training workshops, group coaching and individual mentoring focused on improved understanding of data quality, management, analysis and interpretation for policy. Provision of professional development grants and laptops provided incentives for sustained engagement with the programme on top of participants’ usual workloads.

Unavoidable disruptions included changes to procedures for obtaining ethical approval, political instability, emergence of SARS-CoV-2, and competing demands on participants’ time throughout the project. Scheduling training workshops to maximise attendance proved complicated as health agencies experienced intense workload or important deadlines at different times. Participants felt anxious about letting down their ORCA working group members when they had to prioritise their work responsibilities and indeed, resentment emerged within some groups. However, this also illustrates how seriously participants took their commitment to ORCA activities, and their personal investment in the thematic group work dynamics and outputs.

The ‘embeddedness’ of activities was highlighted as a notable strength of the programme. Both structure and content of ORCA centred around improving RHIS data functionality, leading participants through both desk-based and field investigations of quality and use of RHIS indicators. ‘On the job’ capacity building also allowed for longer-term, continuous support rather than stand-alone training workshops without follow-up on transfer of new skills into the workplace. Managers also appreciated that key staff were not absent for long periods.

We observed early evidence at output level that participants’ knowledge and skills were enhanced in ways they could apply to their roles, and might influence work culture within their regular teams. ORCA’s design helped forge new personal and professional connections by bringing together staff from vertically structured government agencies. Among participants were several team leads and acting directorate heads. Fifteen directorates were reached, representing a potential critical mass for sustainability of new practices. A similar operational research approach to capacity development in Pakistan that trained a comparable number of people found participants maintained active engagement with research and inspired others to enrol in subsequent cohorts [21].

It is still too early to determine whether ORCA was able to influence institutional working cultures that support evidence-based decision-making at policy level [22]. Clearly capacity strengthening needs to go beyond training individuals or even teams to reform of the wider health system [5]. RHIS functionality has previously been characterised as having technical, behavioural and organisational determinants [23]. The ORCA project focused on behavioural aspects, specifically the motivation, skills, and confidence necessary for data use competence. According to Nutley and Reynolds (2013), such competence includes data analysis, interpretation, synthesis, and presentation, all of which formed the core of our activities [24]. Nonetheless, a weakness of our approach is its inability to address some of the more deep-seated structural problems within the health system, including high staff attrition, which has been shown to limit effectiveness of capacity development efforts [4]. We attempted to address this by ‘saturating’ the directorates across the Ethiopian Ministry of Health and its two aligned agencies and providing incentives for staff to engage meaningfully with the programme. Ultimately, however, internal motivators will need to be found to ensure longer-term sustainability and this is likely to require long-term domestic investment and political commitment [25].

Finally, we acknowledge the potential positive bias introduced to our interpretation of ORCA’s effects given our role in ORCA’s design, implementation and documentation [26]. The use of an independent qualitative researcher for conducting in-depth interviews who was unknown to respondents prior to the first interview round likely increased interviewees’ willingness to speak openly about their perceptions, evidenced by their inclusion of negative impressions. We also interpreted our quantitative and qualitative data together as a team, seeking to triangulate across data sources, types of respondents, and agencies to identify where there was overall agreement on benefits and limitations of ORCA [27].

## Conclusions

Strengthening health system capacity remains challenging, despite its increasingly recognised importance. Growing reliance on RHIS data for programming and policy require that public health professionals gain key competencies in diagnosing and improving data quality as well as increasing local data use, moving beyond compilation and onward reporting toward comprehensive analysis and interpretation. ORCA provides one example for how public health analysts across diverse government health agencies can be trained and mentored within the workplace. Despite implementation challenges, ORCA appeared to enhance individual skills and cross-agency team work, with potential relevance to similar health system contexts. Ideally, this process should be implemented in parallel with efforts to address common structural problems such as high staff turnover, inadequate digital infrastructure, and insufficient government ownership.

## Paper context

Routine health information systems in low-income countries are weak and underutilised. We present the Ethiopian Ministry of Health’s tripartite co-designed ORCA capacity development project intending to reflect on it as a case study. We describe the project’s development, implementation, experiences and perceptions. Despite implementation challenges, the project showed enhanced individual skills that could be applied to existing work practices and structures, with potential for broader impact and relevance to similar health system contexts.
